# Supplementary material for: Pathogenicity Analysis of a Novel Variant in GTPBP3 Causing Mitochondrial Disease and Systematic Literature Review
Source: Genes (Basel). 2023 Feb 22;14(3):552. doi: 10.3390/genes14030552 (PMC10048406; doi:10.3390/genes14030552)
Supplement: Supplementary file 1 [file genes-14-00552-s001.zip › genes-2150581-supplementary.pdf]

## Supplemental Figures and Tables

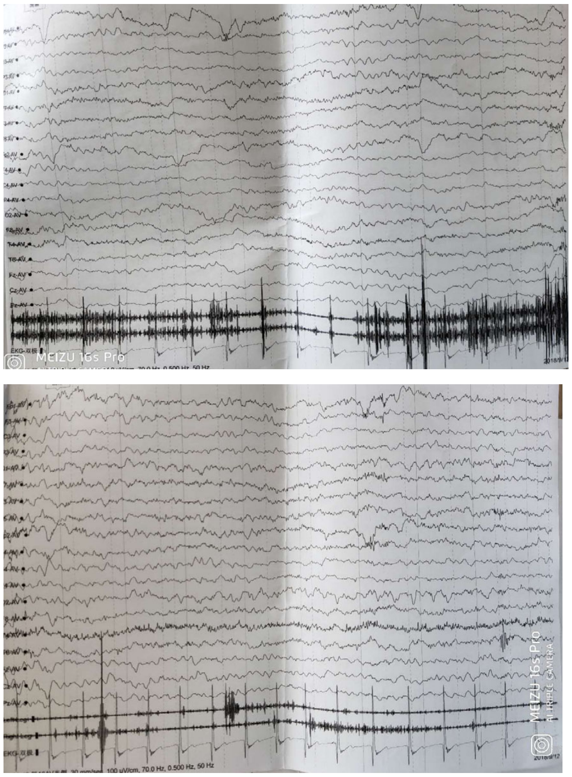

**Figure S1.** Electroencephalogram (EEG) of patient at six years old.

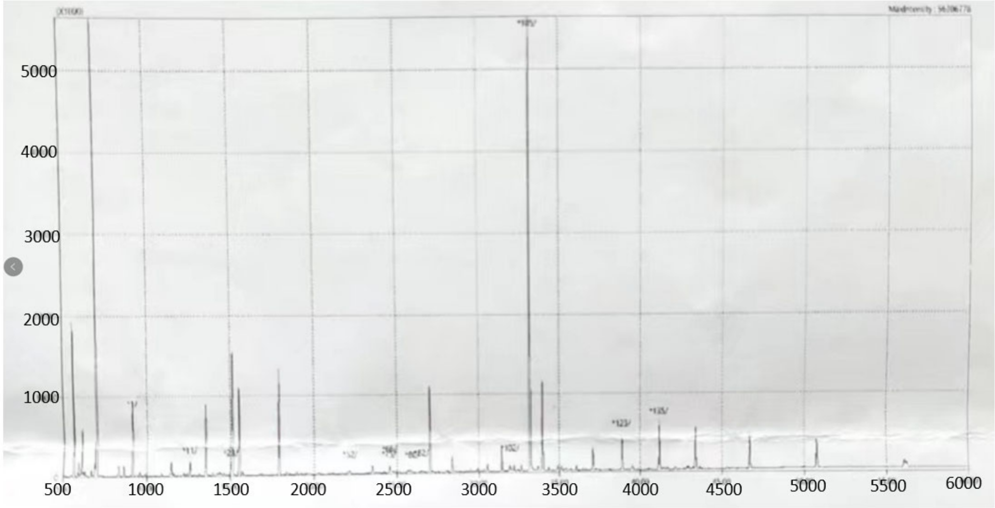


**Figure S2.** Chromatogram for the organic acids at three year of age.

**Table S1.** Clinical and genetic characteristics of patients carrying GTPBP3 variants.

| Patient ID | Mutation type | Exon/ Intron | cDNA | Protein level | Inheritance | Gender | AO | Outcomes; cause of death | TTE | Brain MRI | Plasma lactate | Other phenotypes | Reference |
|---|---|---|---|---|---|---|---|---|---|---|---|---|---|
| Severe type | | | | | | | | | | | | | |
| #83904[*,a] | homozygote | Exon 1 | c.32_33delinsGTG | p.Gln11Argfs*98 | UNK | F | 1 week | died 9 months; cardiac insufficiency with arrhythmia | DCM | UNK | 20 mmol/L ↑ | WPW; cardiogenic shock; metabolic acidosis | Kopajtich et al. (2014) [3] |
| #83905[*,a] | homozygote | Exon 1 | c.32_33delinsGTG | p.Gln11Argfs*98 | UNK | F | birth | died 10 days; cardiac insufficiency | DCM | UNK | UNK | WPW; cardiogenic shock; metabolic acidosis | Kopajtich et al. (2014) [3] |
| #81471 | homozygote | Exon 4 | c.424G>A | p.Glu142Lys | UNK | M | 4 weeks | died 5 weeks; acidosis | HCM (concentric LVH) | hyperintensities in subthalamic nuclei | 11 mmol/L ↑ | weight loss; hypothermia; jaundice; recurrent apnea; metabolic acidosis | Kopajtich et al. (2014) [3] |
| #76671 | homozygote | Intron 5 | c.665-2delA | p.Ala222Gly; p.Asp223_Ser270del | UNK | M | birth | died 10 months; CHF | HCM (RVH) | bilateral hyperintensities in thalamus | 5.2 mmol/L ↑ | WPW; hypotonia; FTT; metabolic acidosis | Kopajtich et al. (2014) [3] |
| #75191 | homozygote | Exon 8 | c.1009G>C | p.Asp337His | UNK | F | birth | died 1 day; asystolia | HCM (apical RVH) | UNK | 23 mmol/L ↑ | Kussmaul breathing; stridor; hyporeactivit; poor feeding; hypotonia; | Kopajtich et al. (2014) [3] |

| | | | | | | | | | | | | | |
|---|---|---|---|---|---|---|---|---|---|---|---|---|---|
| | | | | | | | | | | | | bradycardia | |
| #1 | Compound heterozygote | Exon 4<br>Exon 4 | c.413C>T<br>c.509_510del | p.Ala138Val<br>p.Gln170Glyfs*42 | paternal<br>maternal | M | 17 h | died 5 days; CHF | normal | UNK | 26 mmol/L ↑ | hypothermia; stridor; hyporeactivit; respiratory failure; cardiogenic shock; metabolic acidosis | Yan HM et al. (2021) [4] |
| #72425 | Compound heterozygote | Exon 4<br>Exon 6<br>Exon 7 | c.484G>C<br>c.673G>A<br>c.964G>C | p.Ala162Pro<br>p.Glu225Lys<br>p.Ala322Pro | maternal<br>paternal<br>paternal | F | 3.5 months | died 8 months; CHF | DCM | UNK | 23.3 mmol/L ↑ | poor feeding; FTT; hyporeactivity | Kopajtich et al. (2014) [3] |
| Mild type | | | | | | | | | | | | | |
| #75168* | homozygote | Exon 6 | c.770C>A | p.Pro257His | UNK | F | 2 years | alive 5 years | UNK | bilateral hyperintensities in thalamus and extending to the mesencephalon | >10 mmol/L ↑ | developmental delay; epileptic seizures; intellectual disability | Kopajtich et al. (2014) [3] |
| Patient No.24 | homozygote | Exon 7 | c.836C>T | p.Pro279Leu | UNK | F | 3 weeks | alive 10 years | normal | Delayed myelination | UNK | Mental motor retardation; epileptic seizure; hearing disability; thrombocytopenia | Eimas et al. (2019) [13] |
| #82790 | Compound heterozygote | Exon 1 | c.8G>T | p.Arg3Leu | UNK | F | 1 year | alive 2 years | normal | bilateral hyperintensities | 5.7~6.5 mmol/L | developmental delay; epileptic seizures; | Kopajtich et al. |

| | | | | | | | | | | | | | |
|---|---|---|---|---|---|---|---|---|---|---|---|---|---|
| | | Exon 7 | c.934_957del | p.Gly312_Val319del | UNK | | | | | in the putamen and weakly also in the anterior thalamus | ↑ | hypotonia | (2014) [3] |
| #3 | Compound heterozygote | Exon 4 | c.424G>A | p.Glu142Lys | maternal | F | 1 year | alive 3 years | HCM (LVH) | bilateral lesions in brain stem, thalamus and dentate body of the cerebellum | 4.26~16 mmol/L ↑ | developmental delay; intellectual disability; fatigability | Yan HM et al. (2021) [4] |
| | | Exon 5 | c.689A>C | p.Gln230Pro | paternal | | | | | | | | |
| #66143 | Compound heterozygote | Exon 4 | c.476A>T | p.Glu159Val | UNK | M | 2 years | alive 5 years | HCM | UNK | UNK (lactic acidosis) | sudden respiratory failure; CHF | Kopajtich et al. (2014) [3] |
| | | Exon 7 | c.964G>C | p.Ala322Pro | UNK | | | | | | | | |
| #2 | Compound heterozygote | Exon 4 | c.544G>T | p.Gly182* | paternal | F | 1 year | alive 3 years | UNK | bilateral lesions in the midbrain, thalamus and dentate body of the cerebellum | 7.7~14 mmol/L ↑ | developmental delay; hypotonia | Yan HM et al. (2021) [4] |
| | | Exon 5 | c.689A>C | p.Gln230Pro | maternal | | | | | | | | |
| #C1 | Compound heterozygote | Exon 5 | c.689A>C | p.Gln230Pro | maternal | F | 3 years | alive 3 years | HCM (LVH) | abnormal symmetry signal of bilateral cortical spinal tract | 29 mmol/L ↑ | respiratory failure; myocardial damage; stroke-like syndrome | Yang Q et al. (2021) [15] |
| | | Exon 8 | c.1073delG | p.Gly358Glufs*16 | paternal | | | | | | | | |
| Our Patient | Compound heterozygote | Exon 5 | c.689A>C | p.Gln230Pro | paternal | M | 3 days | alive 10 years | HCM (LVH) | abnormal signal of bilateral thalamus | 3-11.6 mmol/L ↑ | mental and motor retardation; selt-limitd epileptic seizures; abnormal visual | Our Patient |
| | | Exon 7 | c.1102dupC | p.Arg368Profs*22 | maternal | | | | | | | | |

| | | | | | | | | | | | | | |
|---|---|---|---|---|---|---|---|---|---|---|---|---|---|
| | | | | | | | | | | | | development | |
| #C2 | Compound heterozygote | Exon 5 | c.689A>C | p.Gln230Pro | paternal | F | 9 years | alive 17 years | HCM (LVH) | UNK | 5.53 mmol/L ↑ | heart failure; intellectual developmental delay; abdominal pain; abdominal distention | Zhao XX et al. (2021) [14] |
| | | Exon 9 | c.1280delC | p.Pro427Argfs*3 | maternal | | | | | | | | |
| #49665[*,b] | Compound heterozygote | Exon 9 | c.1291dupC | p.Pro431Argfs*87 | UNK | M | 10 years | alive 14 years | HCM (LVH) | Lactate peaks in parietal and precentral cortex | 3~7 mmol/L ↑ | mild intellectual disability; fatigability; limited vision; slight dyspnea with climbing stairs | Kopajtich et al. (2014) [3] |
| | | Exon 9 | c.1375G>A | p.Glu459Lys | UNK | | | | | | | | |
| #36349[*,b] | Compound heterozygote | Exon 9 | c.1291dupC | p.Pro431Argfs*87 | UNK | M | UNK | alive 17 years | HCM | Lactate peaks in parietal and precentral cortex | UNK | mild intellectual disability; fatigability; limited vision; slight dyspnea with climbing stairs | Kopajtich et al. (2014) [3] |
| | | Exon 9 | c.1375G>A | p.Glu459Lys | UNK | | | | | | | | |

Abbreviations are as follows: AO, age of onset; TTE, transthoracic echocardiography; MRI, magnetic resonance imaging; UNK, unknown; CHF, congestive heart failure; DCM, dilated cardiomyopathy; HCM, hypertrophic cardiomyopathy; LVH/RVH, left/right ventricular hypertrophy; WPW, Wolff-Par-kinson-White syndrome; FTT, failure to thrive.

NCBI reference sequence: NM_032620.4.

[*]They have consanguineous parents.

[a,b]These individuals are siblings.

**Table S2.** Distribution of homozygous missense variant in coding region of *GTPBP3* from COXPD23 patients and gnomAD across five structural domains.

| DOMAIN | | **GENERAL POPULATIONS** （HOMO） | **CLINICAL SAMPLES** （HOMO） |
|---|---|---|---|
| **TRANSPORT PEPTIDE REGION** | Number within this domain | 57115 | 0 |
| | Number outside this domain | 66324 | 4 |
| | Fisher's test | | 0.1292 |
| **N-TERMINAL REGION** | Number within this domain | 121670 | 1 |
| | Number outside this domain | 1769 | 3 |
| | Fisher's test | | <0.0001**** |
| **CENTRAL HELICAL DOMAIN 1** | Number within this domain | 1 | 0 |
| | Number outside this domain | 123438 | 4 |
| | Fisher's test | | ＞0．9999 |
| **TRME-TYPE G DOMAIN** | Number within this domain | 1768 | 3 |
| | Number outside this domain | 121671 | 1 |
| | Fisher's test | | <0.0001**** |
| **CENTRAL HELICAL DOMAIN 2** | Number within this domain | 1714 | 0 |
| | Number outside this domain | 121725 | 4 |
| | Fisher's test | | >0.9999 |

**Table S3.** Distribution of variants in GTPBP3 from severe types and mild types across five structural domains.

| Domain | | Total | | LOFVs | | Missense variants | |
|---|---|---|---|---|---|---|---|
| | | severe types | mild types | severe types | mild types | severe types | mild types |
| **Transport peptide region** | Number within this domain | 1 | 1 | 1 | 0 | 0 | 1 |
| | Number outside this domain | 8 | 13 | 2 | 5 | 6 | 8 |
| | Fisher's test | | **>0.9999** | | **0.3750** | | **>0.9999** |
| **N-terminal region** | Number within this domain | 2 | 3 | 0 | 0 | 2 | 3 |
| | Number outside this domain | 7 | 11 | 3 | 5 | 4 | 6 |
| | Fisher's test | | **>0.9999** | | **>0.9999** | | **>0.9999** |
| **Central helical domain 1** | Number within this domain | 3 | 4 | 1 | 2 | 2 | 2 |
| | Number outside this domain | 6 | 10 | 2 | 3 | 4 | 7 |
| | Fisher's test | | **>0.9999** | | **>0.9999** | | **>0.9999** |
| **TrmE-type G domain** | Number within this domain | 4 | 3 | 2 | 1 | 2 | 2 |
| | Number outside this domain | 5 | 11 | 1 | 4 | 4 | 7 |
| | Fisher's test | | **0.3630** | | **0.4643** | | **>0.9999** |
| **Central helical domain 2** | Number within this domain | 0 | 3 | 0 | 2 | 0 | 1 |
| | Number outside this domain | 9 | 11 | 3 | 3 | 6 | 8 |
| | Fisher's test | | **0.2530** | | **0.4643** | | **>0.9999** |
